# The (pro)renin receptor. A decade of research: what have we learned?

**DOI:** 10.1007/s00424-012-1105-z

**Published:** 2012-04-28

**Authors:** Manne Krop, Xifeng Lu, A.H. Jan Danser, Marcel E. Meima

**Affiliations:** Division of Vascular Medicine and Pharmacology, Department of Internal Medicine, Erasmus MC, Dr. Molewaterplein 50, 3015 GE Rotterdam, The Netherlands

**Keywords:** Angiotensin, ATP6AP2, Prorenin, Vacuolar H^+^-ATPase, Wnt

## Abstract

The discovery of a (pro)renin receptor ((P)RR) in 2002 provided a long-sought explanation for tissue renin–angiotensin system (RAS) activity and a function for circulating prorenin, the inactive precursor of renin, in end-organ damage. Binding of renin and prorenin (referred to as (pro)renin) to the (P)RR increases angiotensin I formation and induces intracellular signalling, resulting in the production of profibrotic factors. However, the (pro)renin concentrations required for intracellular signalling in vitro are several orders of magnitude above (patho)physiological plasma levels. Moreover, the phenotype of prorenin-overexpressing animals could be completely attributed to angiotensin generation, possibly even without the need for a receptor. The efficacy of the only available putative (pro)renin receptor blocker handle region peptide remains doubtful, leading to inconclusive results. The fact that, in contrast to other RAS components, (P)RR knock-outs, even tissue-specific, are lethal, points to an important, (pro)renin-independent, function of the (P)RR. Indeed, recent research has highlighted ancillary functions of the (P)RR as an essential accessory protein of the vacuolar-type H^+^-ATPase (V-ATPase), and in this role, it acts as an intermediate in Wnt signalling independent of (pro)renin. In conclusion, (pro)renin-dependent signalling is unlikely in non-(pro)renin synthesizing organs, and the (P)RR role in V-ATPase integrity and Wnt signalling may explain some, if not all of the phenotypes previously associated with (pro)renin-(P)RR interaction.

## Introduction

The renin–angiotensin system (RAS) is a hormonal system that regulates blood pressure by influencing vasomotor tone and salt and fluid retention. The central regulator in this cascade is renin, which catalyzes the rate-limiting step in the formation of angiotensin (Ang) II, the main effector peptide of the system. Surprisingly, the inactive precursor of renin, called prorenin, also circulates in blood, at concentrations that are ten times higher, and in diabetes and pregnancy even up to 100 times higher. Interestingly, the elevated plasma prorenin levels correlate with, and even precede, the microvascular complications of diabetes, although renin levels remain in the low–normal range [25, 65]. As these patients respond well to RAS blockade, it seems likely that prorenin may somehow contribute to Ang II generation at tissue sites. However, since the kidney is the only known site where prorenin-to-renin conversion occurs [60], a longstanding question is how prorenin might display activity extrarenally. This appeared to be solved in 2002 with the discovery of a receptor that could bind and activate prorenin [76]. This receptor also binds renin and is therefore now known as the (pro)renin receptor ((P)RR). Following its discovery, intense research started into its function, which had some initial success stories, but is recently moving more and more to prorenin-independent functions. Now, after a decade of (P)RR research, it is time to make up the score for this receptor: Does it deserve its name, and is it still a potential drug target in cardiovascular disease?

## The search for a receptor

Renin is a member of the family of aspartic proteases. These proteases have two highly conserved aspartic residues in the active cleft, which work optimally at acidic pH. In this aspect, renin is an exception, as it cleaves its substrate angiotensinogen at neutral pH in blood plasma. Renin is produced in juxtaglomerular (JG) cells as prorenin, which contains a 43-amino-acid prosegment that covers the active cleft. The prosegment inactivates the protease and thereby prevents intracellular proteolysis, but it is also thought to be important for folding, stability, and intracellular sorting of proteases [51]. The prosegment is removed in lysosome-like structures that subsequently store renin in dense vesicles [91]. Renin is released from JG cells in a regulated manner, in response to stimuli like hypotension and low Na^+^. Remarkably, the inactive prorenin is also secreted directly into the bloodstream. This occurs in a constitutive manner, and prorenin is not stored.

After a bilateral nephrectomy, renin rapidly disappears from plasma [56]. Prorenin levels also rapidly decline, but then stabilize due to prorenin synthesis at extrarenal sites, like the adrenal gland, the testes, the ovaries, placenta, and the eyes [55]. Recent studies suggest that the collecting duct in the kidney is an alternative source of prorenin [82], possibly contributing to the elevated prorenin levels in diabetics [49]. Interestingly, although aortic (and cardiac) renin also disappeared post-nephrectomy, their disappearance occurred much more slowly than that of plasma renin [23, 98]. This suggests that there are binding places that can retain renin. Such binding places are in agreement with the fact that the majority of Ang I formation occurs at tissue sites, e.g., in the vessel wall. The first such receptor that was identified was the mannose-6-phosphate receptor, which is identical to the insulin-like growth factor II (IGFII) receptor and hence is called M6P/IGFII receptor [53]. M6P/IGFII receptors are present on neonatal cardiac myocytes and fibroblasts [85], as well as on human endothelial cells [100] and vascular smooth muscle cells [4]. This receptor binds phosphomannosylated proteins like renin and prorenin with high affinity, and both are internalized rapidly. Although prorenin was converted to renin intracellularly following internalization, there is no evidence that this resulted in either intracellular or extracellular Ang I generation [85, 100]. In fact, the prorenin-to-renin conversion was found to be part of a slow intracellular degradation process, and the M6P/IGFII receptor is therefore now thought to be a clearance receptor for renin and prorenin [22].

Nguyen and co-workers identified a second receptor in 2002, called the (P)RR, that binds both renin and prorenin without internalization [76]. The (P)RR induces a fivefold increase in the activity of bound renin by increasing the affinity of renin for the oxidized form of its substrate angiotensinogen [109]. Binding of prorenin to the (P)RR enables prorenin to undergo a conformational change that allows it to gain complete enzymatic activity without proteolytical removal of the prosegment [4, 5, 72, 76]. Binding possibly involves a region in the prosegment, and on this basis, an antagonist (the so-called handle region peptide (HRP)) has been developed [40, 95].

## The many faces of the (pro)renin receptor

**Fig. 1:**
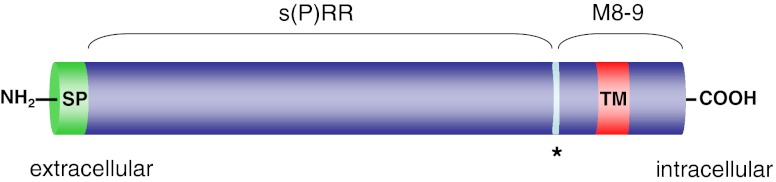
Predicted domain structure and proteolytic fragments of the (P)RR. A putative furin cleavage site is indicated by an asterisk. Abbreviations: SP, signal peptide; TM, transmembrane domain

The (P)RR is a 350-amino-acid, ubiquitously expressed transmembrane protein that binds (pro)renin to a large N-terminal extracellular domain (Fig. 1) [76]. The (pro)renin binding domain is only conserved in vertebrates, which probably means that this function of the (P)RR was acquired during vertebrate evolution [30]. Both prorenin and renin can bind to purified (P)RR with affinities in the nanomolar range [4, 72, 76]. However, binding studies in rat vascular smooth muscle cells that overexpress the human (P)RR have shown that prorenin binds with higher affinity, suggesting that prorenin might be the endogenous ligand for the (P)RR [4]. The (pro)renin-binding domain is followed by a carboxyterminal half that is comprised of the rest of the extracellular domain, a single transmembrane domain, and a short intracellular tail that can form dimers [107]. The carboxyterminal part is conserved among all metazoans [30]. It was previously purified from chromaffin granules as an 8.9-kD accessory protein (M8-9) of the vacuolar-type H^+^-ATPase (V-ATPase) and designated ATP6AP2 (ATPase, H^+^-transporting, lysosomal accessory protein 2) [64]. This would imply that the (P)RR can be proteolytically processed. Indeed, Cousin et al. [18] found a 28-kD soluble form of the (P)RR, designated s(P)RR, in the conditioned medium of several cell types [18]. This form was, however, absent in the medium of LoVo cells, which are devoid of the serine protease furin. In addition, the formation of the s(P)RR was also absent from the medium of CHO cells after furin inhibition, and recombinant furin could directly cleave the (P)RR in vitro [18]. Furthermore, a putative furin cleavage site is predicted around the expected position in the (P)RR amino acid sequence (Fig. 1), all suggesting that furin is the protease responsible for the generation of s(P)RR. This, however, was recently challenged by Yoshikawa et al. [105], who did find s(P)RR in the medium of LoVo cells and in the medium of vascular smooth muscle cells (VSMCs) after furin inhibition. Instead, these authors identified the metalloprotease ADAM19 as a (P)RR-cleaving protease [105]. s(P)RR secreted by human umbilical vein endothelial cells (HUVECs) and VSMCs appears functional, as it can bind and activate prorenin [7, 105]. Using immobilized renin, Cousin et al. precipitated and immunodetected s(P)RR from human plasma [18]. However, others could only detect s(P)RR in human urine but not plasma [43]. s(P)RR was also found in the urine of Ang II infused rats [32]. Whether there is a physiological function for s(P)RR in angiotensin-generation or signalling is as yet unclear, although some have speculated that s(P)RR itself may act as a ligand for another, yet unknown, receptor [3].

## The (P)RR as a V-ATPase-associated protein

**Fig. 2:**
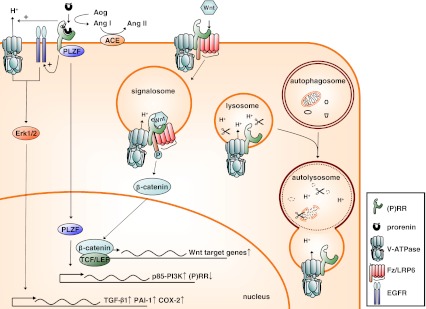
Functions of the (P)RR in (pro)renin activation and signalling (*left*), Wnt signalling (*middle*), and V-ATPase integrity in autophagic digestion (*right*). See text for explanation. Abbreviations used: *ACE*, angiotensin-converting enzyme; *Ang*, angiotensin; *Aog*, angiotensinogen; *COX-2*, cyclooxygenase-2; *EGFR*, epidermal growth factor receptor; *Erk1/2*, extracellular signal-regulated kinase 1/2; *Fz/LRP6*, frizzled/low-density lipoprotein receptor-related protein 6; *PAI-1*, plasminogen-activator inhibitor-1; *PI3K*, phosphoinositide 3-kinase; *PLZF*, promyelocytic leukemia zinc finger protein; *(P)RR*, (pro)renin receptor; *TCF/LEF*, T cell factor/lymphocyte enhancer-binding factor; TGF-β1, transforming growth factor-β1

Recent work has indicated the importance of the association between the (P)RR and V-ATPase for development and cell survival, independent from (pro)renin. V-ATPases are multisubunit proteins that consist of a V_0_ proton-translocation domain, a V_1_ pump domain, and two associated proteins, Ac45 and the (P)RR. V-ATPases are found in virtually all cell types, mostly on the membranes of intracellular compartments, and are important for among others vesicle trafficking, protein degradation, and coupled transport [99]. In some cell types, V-ATPases are also abundantly present at the plasma membrane, for example, in intercalated cells of the collecting duct, where they regulate systemic acid–base homeostasis [103] and in osteoclasts, where they are involved in bone resorption [61]. The (P)RR was shown to colocalize with the V-ATPase at the lumen of intercalated cells and at the Z-disc and dyad in cardiomyocytes [16]. Insertional mutagenesis of the *(P)RR* gene in zebrafish and injection of morpholino RNAs against the *(P)RR* into cleavage-staged *Xenopus* embryos give very similar developmental phenotypes, with larvae and tadpoles that have smaller heads and defects in eye and melanocyte pigmentation [2, 19], similar to the phenotypes found for V-ATPase subunits mutants [2, 19, 77]. Because *(P)RR* knock-out mice are lethal at a very early developmental stage [93], the focus to unravel the (patho)physiological role of the (P)RR has shifted to the generation of tissue-specific knockouts. These studies have indicated an essential role for the (P)RR in V-ATPase integrity. Cardiomyocyte-specific ablation of the *(P)RR* results in a lethal phenotype, with mice dying within 3 weeks of heart failure [52]. Similarly, podocyte-specific *(P)RR* knock-out mice are born at Mendelian rates, but, early in life, develop nephritic syndrome, with severe proteinuria and albuminuria due to progressive glomerular sclerosis, and die within 2–4 weeks of renal failure [68, 79, 84]. The podocytes of these mice show massive foot-process effacements accompanied by alterations in the actin cytoskeleton, whereas the slit diaphragm proteins, nephrin and podocin, have reduced expression and are redistributed to the cytosol [79, 84]. Both (P)RR depleted cardiomyocytes and podocytes are highly vacuolarized and show impaired autophagic degradation (Fig. 2). The autophagy defect is due to deacidification of intracellular vesicles, which is caused by the selective downregulation of V_0_-subunits [52, 79]. These findings show the importance of the (P)RR for V-ATPase assembly, stability, and function, and suggest that more than an accessory protein, the (P)RR is an essential V-ATPase subunit.

## The (pro)renin receptor as a signalling receptor

Apart from prorenin activation, the (P)RR can also act as a signalling receptor for (pro)renin, independent from the formation of Ang I (Fig. 2). Binding of (pro)renin to the (P)RR activates the mitogen-activated protein kinase (MAPK) extracellular signal-regulated kinase 1/2 (Erk1/2) in several cell types, including mesangial cells [37, 76], collecting duct cells [1], VSMCs [6, 31, 62], monocytes [30], and neurons [17]. Activation of Erk1/2 increases cell proliferation and stimulates production of transforming growth factor-β1 (TGF-β1), resulting in the upregulation of profibrotic factors, such as the plasminogen-activated inhibitor-1 (PAI-1), fibronectin, and collagen [37, 38, 106]. Increased expression of the NADPH oxidase isoform 4 [14] may be involved. Prorenin binding also induces the activation of the serine/threonine kinase Akt in VSMCs [62] and of the p38 MAPK–heat-shock protein 27 cascade in cardiomyocytes [86]. The latter cascade regulates actin cytoskeleton dynamics. How does the (P)RR signal towards downstream effectors? The (P)RR shares no homology with known signalling receptors, and amino acid sequence of the short intracellular tail predict no protein–protein interaction domains, however, possible candidates have been identified. Using a yeast two-hybrid system, Schefe et al. showed that the promyelocytic leukemia zinc finger (PLZF) can act as a (P)RR binding protein (Fig. 2) [89, 90]. Prorenin binding induces the translocation of PLZF to the nucleus, where it activates the expression of the p85-subunit of phosphoinositide 3-kinase, resulting in enhanced protein synthesis, cell proliferation, and decreased apoptosis. Whether PLZF is also involved in the activation of other signalling molecules such as Erk1/2 is as yet unknown but seems unlikely as it is a transcriptional regulator rather than a signalling protein. Erk1/2 can be activated by some G-protein-coupled receptors, for example, the angiotensin II type 1 receptor (AT1), through transactivation of growth factor receptors [27]. This may also be true for the (P)RR, as in VSMCs, prorenin-induced transactivation of the epidermal growth factor receptor (EGFR) is required for Erk1/2 and Akt phosphorylation [62]. Erk1/2 activation is also linked to V-ATPase activity, as prorenin-induced Erk1/2 phosphorylation in Madin Darby Canine Kidney (MDCK) cells, a model for collecting duct cells, is inhibited by the V-ATPase inhibitor bafilomycin A1 [1]. Studies in our lab have shown that prorenin directly stimulates plasma membrane-associated V-ATPase activity in MDCK cells (Fig. 2) [63], indicating that the V-ATPase may increase intracellular pH (pH_i_) above a permissive level required for Erk1/2 activation, comparable to Ang II activation of Erk1/2 in rat aorta smooth muscle cells [71]. How the (P)RR activates the V-ATPase is as yet unclear. This could involve increased cycling of V-ATPase-containing vesicles to the membrane by changes in actin cytoskeletal dynamics [20, 86] or, since it directly binds to V-ATPase [19], a conformational change in the complex. In summary, (pro)renin induces multiple signalling pathways via the (P)RR in cell models. Is this also reflected in vivo?

## Lessons from humans

A unique exonic splice enhancer mutation that results in a partly truncated form of the receptor (Δ4-(P)RR) was found in a family with X-linked mental retardation and epilepsy [83]. No display of cardiovascular or renal abnormalities were reported. The (P)RR truncation could still bind and activate renin but was unable to activate Erk1/2. When co-expressed in PC-12 neuronal cells, Δ4-(P)RR acted as a dominant negative, by altering localization of wild-type (P)RR and preventing prorenin-induced Erk1/2 phosphorylation [17].

Genome-wide association studies have identified several single nucleotide polymorphisms that are associated with increased cardiovascular risk. In a Japanese cohort, the intervening sequence 5 + 169C>T polymorphism was significantly and independently related to ambulatory blood pressure in men but not in women [35]. The significant higher ambulatory blood pressure for T-allele carriers was confirmed in Caucasian men for the systolic but not diastolic blood pressure [80]. Renal plasma flow showed no difference between the two alleles. Also, two polymorphisms, one in the promoter region and one in an intron of *(P)RR*, were significantly associated with hypertension in two vascular disease populations of CAD (EUROPA) and cerebrovascular disease (PROGRESS) [8]. In Japanese women, but not men, the risk of lacunar infarction and left ventricular hypertrophy was significantly and independently associated with the +1513A>G polymorphism [36]. The risk in GG women was significantly higher than in women with either AA or AG genotype, although GGs displayed a lower plasma renin activity.

## Lessons from transgenic models

Efforts to study the role of the (P)RR in cardiovascular pathology have been hampered by the fact that *(P)RR* knockout mice are lethal, and even tissue-specific knock-outs, as discussed previously, have a life expectancy that is too short for comprehensive studies [52, 79, 84]. Therefore, several groups have used transgenic murine models in which either the (P)RR or its ligand prorenin are overexpressed. Binding of rat prorenin to human (P)RR (h(P)RR) induces Erk1/2 phosphorylation but not prorenin activation [48], and therefore overexpression of the h(P)RR in rats will yield an Ang-independent phenotype. These rats are characterized by upregulated expression of the proinflammatory factor cyclooxygenase 2 (COX-2) in the kidney and the development of glomerulosclerosis in the absence of hypertension or changes in renal RAS activity [47, 48]. In contrast, rats that overexpress the h(P)RR specifically in smooth muscle cells have elevated blood pressure after 6 months but normal kidney function [10]. The rise in blood pressure may be caused by increased plasma aldosterone levels in these rats. Interestingly, mineralocorticoid receptor blockade has indeed been proven beneficial in heminephrectomized (P)RR transgenic rats [70]. In addition, the *(P)RR* gene receptor polymorphism associated with increased blood pressure in Caucasian men is also associated with increased plasma aldosterone levels [80]. Since prorenin is also secreted and released by the adrenal gland, this could mean that the (P)RR can function as a regulator of intra-adrenal RAS activity. However, direct effects of prorenin via the (P)RR on adrenal aldosterone production could not be demonstrated [46].

**Fig. 3:**
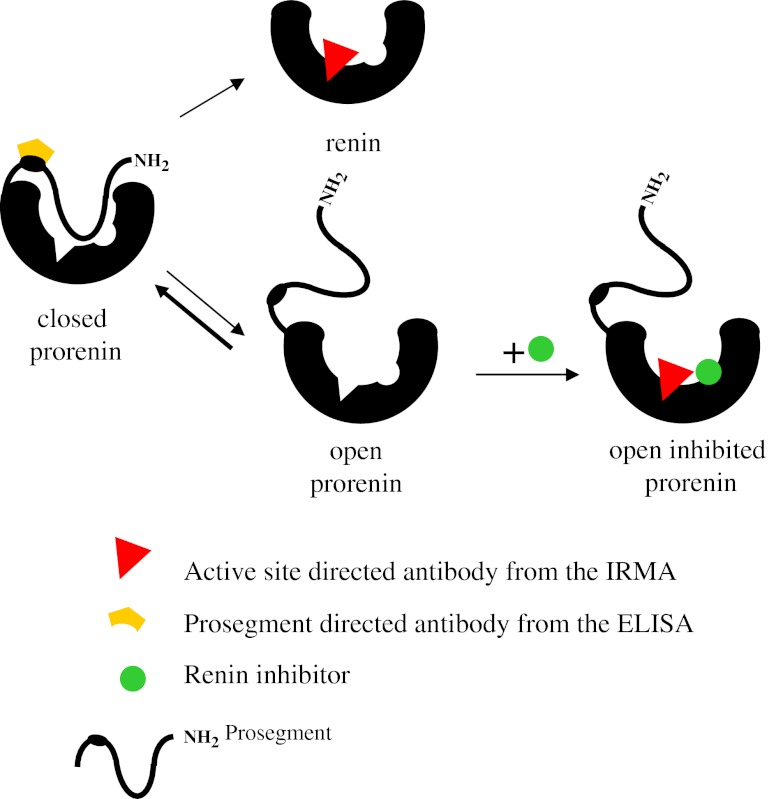
Prorenin detection with different assays. Until recently, measuring prorenin immunologically could only be done using a renin-specific immunoradiometric assay (IRMA). This kit makes use of an antibody recognizing the active site, and therefore, to allow prorenin detection, it first has to be converted to renin by cleaving off the prosegment, e.g., with trypsin. Alternatively, a renin inhibitor can interfere with the equilibrium that exists between ‘closed’ (inactive) and ‘open’ (active) prorenin. Normally, >98 % of prorenin is in the closed conformation. Yet, in the presence of a renin inhibitor, the equilibrium will shift into the direction of the open conformation [5, 26], i.e., exposing the active site, while the prosegment is still attached. Indeed, treatment for 48 h at 4 °C with the renin inhibitor aliskiren (10 μmol/L) [5] fully converts all prorenin molecules to the open conformation, allowing their detection in a renin-specific assay. This approach, as well as the proteolytic approach (cleaving off the prosegment), requires renin to be measured before and after treatment in order to determine the amount of prorenin from the difference between the two measurements. Recently, a new prorenin specific ELISA has been developed, making use of an antibody directed against the prosegment [58], allowing direct prorenin measurement without pretreatment. The method is fast, and the results are very consistent with those obtained with the renin IRMA measurements

Although prorenin overexpression was initially reported to induce renal and cardiac pathology without increasing blood pressure [102], this could not be confirmed in later studies by the same group [11] and others [69, 81]. These later studies did show a rise in blood pressure following prorenin overexpression. Blood pressure normalized by concurrent RAS inhibition or expression of an active-site mutated prorenin [69], indicating that the hypertension was Ang II-dependent. Indeed, plasma renin decreased after a 200-fold increase in prorenin concentration [81], supporting intrarenal Ang II formation and a negative feedback effect on endogenous renin release. As a small percentage (≈2 %) of plasma prorenin is always in the open form, i.e., capable of generating Ang I (Fig. 3), a 200-fold increase of plasma prorenin concentration leads to a substantial increase in Ang I-generating activity, even without the help of a receptor. This should be taken into account when considering prorenin-dependent angiotensin generation.

## Lessons from animal models

Several studies have focused on the (P)RR contribution to the cardiovascular complications associated with diabetes mellitus. (P)RR expression is upregulated in the kidneys from patients [97] and rats [67] with diabetic nephropathy. To study the involvement of the (P)RR in these models, the peptidic (P)RR blocker called HRP was developed. HRP binds to immobilized (P)RR in the nanomolar range and partly displaces bound renin and prorenin [73, 74]. HRP infusion normalized renal Ang II levels and prevented the development of or even reversed existing nephropathy in diabetic rats [40, 96]. HRP also reduced glomerulosclerosis in diabetic mice deficient for the AT1a receptor [42] and in (P)RR transgenic rats [48], indicating that some of the effects of (P)RR activation in the kidney are independent of renal Ang II generation. Similarly, retinal inflammation in diabetic mice was not only reduced by AT1 receptor blockade (ARB) in wild-type mice [75] but also by HRP in AT1a receptor-deficient mice [87]. Also, in hypertensive models, beneficial effects were found for HRP, for example, in spontaneously hypertensive rats (SHRs) on a high salt diet, where it improved kidney and left ventricular function and decreased left ventricular mass and fibrosis [94]. In stroke-prone SHRs on a high salt diet, HRP decreased cardiac Ang II levels and attenuated cardiac fibrosis, without effects on plasma RAS activity [41]. However, the efficacy of HRP has been controversial. HRP had no effect on blood pressure or renal injury in hypertensive Goldblatt rats [4] or double-transgenic rats that overexpress prorenin and angiotensinogen [31]. Furthermore, HRP, at concentrations up to 1 μmol/L, failed to block (pro)renin binding in rat VSMCs overexpressing human (P)RR [4], or Erk1/2 phosphorylation in human VSMCs [31] and U937 monocytes [30] exposed to prorenin. Moreover, even at 100 μmol/L, HRP blocked renin and prorenin binding only partly [73]. Indeed, Leckie et al. [59] were unable to find a specific binding place for HRP on HUVECs.

## The (P)RR and RAS inhibition

Renin secretion is under tight control of Ang II levels through a negative feedback loop, and therefore RAS blockade causes a reactive rise in plasma renin and, to a lesser extent, plasma prorenin concentrations. Direct renin inhibition appeared to increase the renin concentration even more than ACE inhibition (ACEi) or ARB, due to the detection of prorenin as renin [57] (Fig. 3) and decreased renin/prorenin clearance [5]. Hence, RAS inhibition (and in particular renin inhibition) could in theory have adverse effects through activation of the (P)RR. It has been suggested [28] that this may explain the increased risk of cardiovascular death in hemodialysis patients that undergo dual therapy with ACEi and ARB as compared with a single RAS blocker [12]. However, this is as yet speculative and not corroborated by other studies so far [21]. In cells, (pro)renin stimulation of PLZF downregulates the expression of the *(P)RR* gene through a short negative-feedback loop [89], which is unopposed by the renin inhibitor aliskiren [29, 31, 90]. Could decreased (P)RR expression explain the absence of (P)RR-dependent effects during RAS inhibition? In diabetic TG(mRen-2) rats, aliskiren reduced (P)RR expression in glomeruli, tubules, and cortical vessels [29]. However, the (P)RR was upregulated in the clipped kidney of Goldblatt hypertensive rats treated with a vasopeptidase inhibitor, when both plasma (pro)renin and renal renin are strongly increased [54]. In addition, other factors than PLZF, for example, COX-2 in diabetes [13, 39], can regulate (P)RR expression, so, clearly, (P)RR expression is the result of more factors than just prorenin.

Is there an additional benefit of add-on therapy, i.e., (P)RR inhibition on top of ACEi or ARB? In an experimental model, using cultured glomeruli from rats with anti-Thy-1-induced glomerulonephritis, addition of (P)RR siRNA to an already maximal dose of the ACE inhibitor enalaprilate further decreased fibrotic factor production compared with single therapy [108]. However, in SHR rats with add-on therapy of HRP different results were obtained. While HRP combined with an ACEi decreased heart weight and urinary protein excretion more than with ACEi alone [92], it reversed the beneficial effects of aliskiren on cardiac hypertrophy, blood pressure, and coronary circulation [101]. Again, the results with HRP have to be considered with care and raise the necessity for other (P)RR blockers that prove their efficacy and specificity in both in vitro and in vivo models.

## The (P)RR: a physiological (pro)renin receptor?

If anything has become clear from the in vivo models, it is that, in spite of the promise, the results have been underwhelming. Models with prorenin overexpression were found to display an Ang II-dependent phenotype [11, 69, 81], which can be explained, at least in part, by the well-known fact that a small part of prorenin is present in its open active form in plasma. Does (pro)renin–(P)RR interaction ever occur in vivo? The dissociation constants for renin and prorenin range between 1 and 20 nM, depending on the use of membrane fractions of (P)RR overexpressing cells [4, 76], or immobilized receptors [72, 74]. With a few exceptions, most in vitro experiments that show (P)RR-dependent signalling use (pro)renin concentrations in this range. However, plasma renin and prorenin concentrations are in the picomolar range (≈0.5 and 5 pmol/L, respectively) [24]. Plasma renin concentration can be increased up to 100-fold by, among others, Bartter’s syndrome, renal artery stenosis or RAS inhibition, and prorenin concentration are increased by maximally five- to tenfold in pregnancy and in patients with diabetes [88]. Even the excessive prorenin levels present in transgenic models are still an order of magnitude below those applied in vitro. A recent detailed study by Batenburg et al. [6] investigated the effect of different concentrations of human prorenin and renin in VSMCs that overexpress the human (P)RR. In this model, 4 nmol/L prorenin induced DNA synthesis and PAI-1 production only in the presence of angiotensinogen, whereas 20 nmol/L prorenin was required to achieve angiotensin-independent phosphorylation of Erk1/2, which is respectively ~800- and ~4,000-fold higher than normal. Renin induced DNA synthesis, Erk1/2 phosphorylation, and PAI-1 release at 1 nmol/L (~2,000-fold higher than normal) both in human (P)RR expressing and wild-type cells. As plasma concentrations reflect the extracellular fluid concentration, the prorenin levels in prorenin transgenic animals might thus, at most, result in some modest (P)RR-dependent angiotensin generation, and direct activation of the (P)RR by these levels still seems impossible.

From this point of view, (pro)renin–(P)RR interaction in wild-type animals (including humans under pathological conditions) seems only possible in (pro)renin synthesizing organs like the kidney. (P)RR expression is upregulated, and (pro)renin is secreted in the collecting duct of the kidney under diabetic conditions [49, 67]. In contrast to most other cell types, the (P)RR is abundantly present at the plasma membrane of the intercalated cells of the collecting duct [1]. Since Erk1/2 phosphorylation is already evident at low picomolar concentrations in MDCK cells [1], as well as mesangial cells [37], it is possible that a sufficient number of receptors are occupied in the collecting duct to elicit (P)RR-dependent signalling. The ovaries secrete prorenin as well, and, although the prorenin concentrations in follicular fluid are on average only ≈10 times higher than in plasma, it seems to affect follicular development and oocyte maturation, as the follicular prorenin concentration correlated inversely with follicular atresia [44]. Also, prorenin, secreted from the chorion leave, in the gestational sac during the first trimester can reach up to 1 μg/mL [45], which could be high enough for (P)RR stimulation and has been suggested to play a role in fetal and embryonic development [45].

## (Pro)renin-independent functions for the (P)RR in Wnt signalling

The interaction between the (P)RR and the V-ATPase is not only important for V-ATPase integrity but for signal transduction as well. The *(P)RR* gene was identified in human embryonic kidney cells in an siRNA library screen designed to find previously unknown components of the canonical Wnt/β-catenin signalling pathway [19]. The canonical Wnt/β-catenin signalling pathway is important for embryonic development and tissue homeostasis and has been implicated in certain pathologies, such as cancer and diabetes. Binding of Wnt to frizzled/low-density lipoprotein receptor-related protein 6 (Fz/LRP6) complex results in stabilization of β-catenin, which then translocates to the nucleus where it complexes with the T cell factor/lymphocyte enhancer binding factor (TCF/LEF) to induce the expression of Wnt target genes [15, 66]. Wnt/β-catenin signalling requires the internalization of the Fz/LRP6 complex and subsequent acidification of the signalling endosomes by V-ATPases. Acidification of the endosomes requires the presence of the (P)RR, which acts as a physical adaptor between the Fz/LRP6 complex and the V-ATPase [19]. In the Wnt/planar cell polarity (Wnt/PCP) pathway, which regulates the polarization of cells in the plane of tissues, the (P)RR also binds and targets Fz-receptors and is required for gastrulation in *Xenopus* and orientation of wing hairs and notum bristles in *Drosophila* [9, 34]. Importantly, since (pro)renin is not present in early *Xenopus* embryos [19] and (pro)renin has not been identified in *Drosophila*, these functions of the (P)RR are independent from (pro)renin.

Can some of the phenotypes associated with the (P)RR be explained by changes in Wnt- signalling? In diabetic nephropathy, COX-2 exacerbates the disease by upregulating (P)RR expression in podocytes, which contributes to albuminuria, foot-process effacement, and mesangial matrix expression [13]. Combined with the COX-2 upregulation in (P)RR transgenic mice [47], this suggests a link between the (P)RR, COX-2, and renal injury. However, COX-2 is also a target gene for Wnt/β-catenin signalling [78]. Furthermore, Wnt/β-catenin signalling is increased in podocytes and glomeruli of patients and mouse models with diabetic kidney disease [50]. A balanced amount of Wnt/β-catenin signalling seems required for proper kidney function, as both overexpression and deletion of β-catenin in podocytes leads to podocyte dysfunction and susceptibility to diabetic kidney disease [50]. Importantly, renal Wnt/β-catenin signalling acts downstream of TGF-β1 to induce expression of profibrotic markers, including PAI-1, that contributes to renal fibrosis, podocyte injury, and proteinuria [33, 104]. So it appears that some of the signalling molecules involved in (pro)renin signalling in vitro are also important players in Wnt/β-catenin signalling in renal pathology. Hence, the effects of (P)RR overexpression may also be due to overactivation of the Wnt/β-catenin pathway, independent from (pro)renin. In addition, since Δ4-(P)RR not only inhibits (pro)renin signalling in neurons but also neural growth factor signalling [17], the possibility that the (P)RR is involved in other signalling pathways as well cannot be excluded.

## Conclusions

After 10 years of research, the (P)RR still has a case to make to deserve its name. In spite of the in vitro findings, the evidence in in vivo models argues against a (pro)renin-dependent function for the (P)RR. Models that have high plasma levels of (pro)renin have a phenotype that is entirely dependent on Ang II formation, which can possibly even be explained without the need for the (P)RR. The (pro)renin levels in these models are an order of magnitude higher than in the most severe pathological models but still an order of magnitude lower than the concentrations required in virtually all cell-based models, and thus a (pro)renin–(P)RR interaction outside (pro)renin-synthesizing cells appears impossible. Even though beneficial effects for the putative (P)RR blocker HRP have been reported, this has been contradicted by others, and the efficacy in specificity of HRP is questionable, raising the need for more effective and selective (P)RR blockers. Overexpression of the (P)RR yields conflicting results and raises the question whether the observed effects depend on (pro)renin binding. Deletion of the *(P)RR* gene, even in individual tissues, results in a lethal phenotype. Thus, a balanced amount of (P)RR expression seems vital for normal tissue behavior. The same is true for the Wnt/β-catenin pathway, where too much or too little activity interferes with tissue homeostasis. Thus, the question arises if not (pro)renin binding but altered Wnt/β-catenin signalling is responsible for many, if not most, of the phenotypes of models in which (P)RR overexpression is altered. Clearly, compelling in vivo data are required to address this question. In addition, the (P)RR could have a more universal function as an adaptor protein, therefore another challenge will be to determine whether the (P)RR mediates signal output of other pathways than those initiated by Wnt molecules as well. Consequently, the (P)RR may not be a suitable drug target in cardiovascular and renal diseases. As a role of a (pro)renin–(P)RR interaction in pathophysiology still needs to be proven and the receptor appears an essential component of the V-ATPase, interference with (P)RR function may even be detrimental.
